# Perioperative Management of a Child With Pre-existing Diabetes Insipidus Undergoing Craniopharyngioma Excision: A Case Report

**DOI:** 10.7759/cureus.73518

**Published:** 2024-11-12

**Authors:** Prashant B Lakhe, Chayanika Kutum, Nayana Sabu, Anu Kewlani, Ridhima Sharma

**Affiliations:** 1 Department of Neurosurgery, All India Institute of Medical Sciences, Nagpur, Nagpur, IND; 2 Department of Anaesthesiology, All India Institute of Medical Sciences, Nagpur, Nagpur, IND

**Keywords:** anesthetics, craniopharyngioma, diabetes insipidus, electrolytes, hypothalamus, vasopressin

## Abstract

Craniopharyngiomas are rare tumors arising in the suprasellar area of the brain and are more common in the pediatric age group. Due to the involvement of the hypothalamus, central diabetes insipidus (DI) is usually associated with such lesions. Patients with DI are at risk for significant electrolyte disturbances due to high urine output and the potential for sodium imbalance. Perioperative management of a patient with co-existing DI is challenging due to the imbalances in the fluid and electrolyte status. We reported a rare case of successful intraoperative anesthetic management of a pediatric patient undergoing craniopharyngioma excision with pre-existing central DI. The main anesthetic concerns were intraoperative fluid and electrolyte disbalance, and further risk of hypothalamic damage with the possibility of seizure, hyperthermia, and hemodynamic instability. Intraoperatively, a meticulous fluid management strategy was employed, keeping a strict watch on urine output and serum electrolyte levels. Intraoperative DI was treated with low-dose vasopressin infusion. Intensive monitoring of the fluid and electrolyte status in a patient during craniopharyngioma surgery is of utmost importance. A proper collaborative team effort between the neurosurgeons, neuroanesthesiologists, and the neuroendocrine team is essential for a successful outcome.

## Introduction

Craniopharyngiomas are benign tumors that arise from epithelial remnants of the craniopharyngeal duct, typically located in the sellar or suprasellar regions of the brain. They constitute 5-10% of all pediatric brain tumors, primarily presenting between the ages of 5 and 14 years [1]. Managing craniopharyngiomas usually involves surgical resection, frequently followed by adjuvant radiotherapy [2]. Achieving complete resection while preserving neuroendocrine functions remains challenging [3]. Craniopharyngiomas can cause significant hormonal imbalances due to their proximity to the pituitary gland and hypothalamus. The tumor can damage the hypothalamic-pituitary axis, leading to central diabetes insipidus (DI) [4]. Perioperative management of such patients involves carefully monitoring fluid balance and electrolytes. A pertinent perioperative anesthetic management of intravenous fluids and hormone replacement is crucial. Ours is a case of a 12-year-old child with craniopharyngioma with associated pre-existing DI planned for craniotomy and excision of the lesion. This case report was written as per the CARE (CAse REport) guidelines [5]. Written informed consent for publication was obtained from the child’s parents.

## Case presentation

A 12-year-old female child, weighing 33 kg, belonging to the American Society of Anesthesiologists (ASA) I physical status initially, presented with symptoms of persistent headache and seizures. MRI findings revealed a craniopharyngioma in the suprasellar region (3.7*3.1*2.8 cm) causing compression of the optic chiasma and the third ventricle. The patient underwent craniotomy and tumor excision, following which she developed postoperative DI. She was started on tablet desmopressin 0.5 mg HS. The patient was administered tablet thyroxine in view of secondary hypothyroidism. The anti-epileptic prophylaxis was continued. 

**Figure 1:**
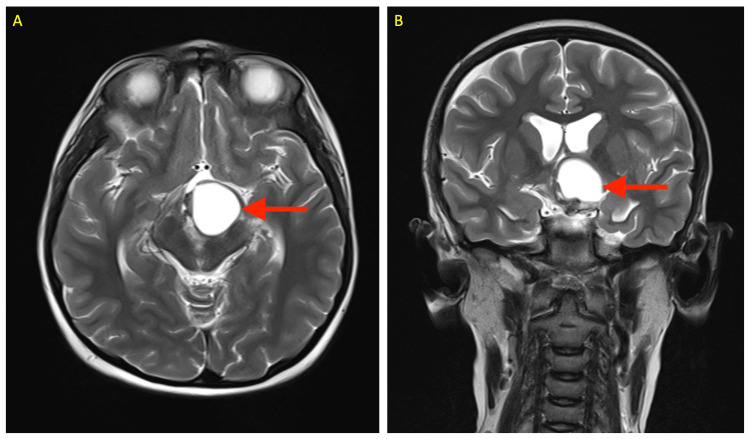
T2 weighted MRI scans showing residual craniopharyngioma in the sellar-suprasellar region of brain (shown by red arrows): (A) Axial view, (B) Coronal view

**Figure 2:**
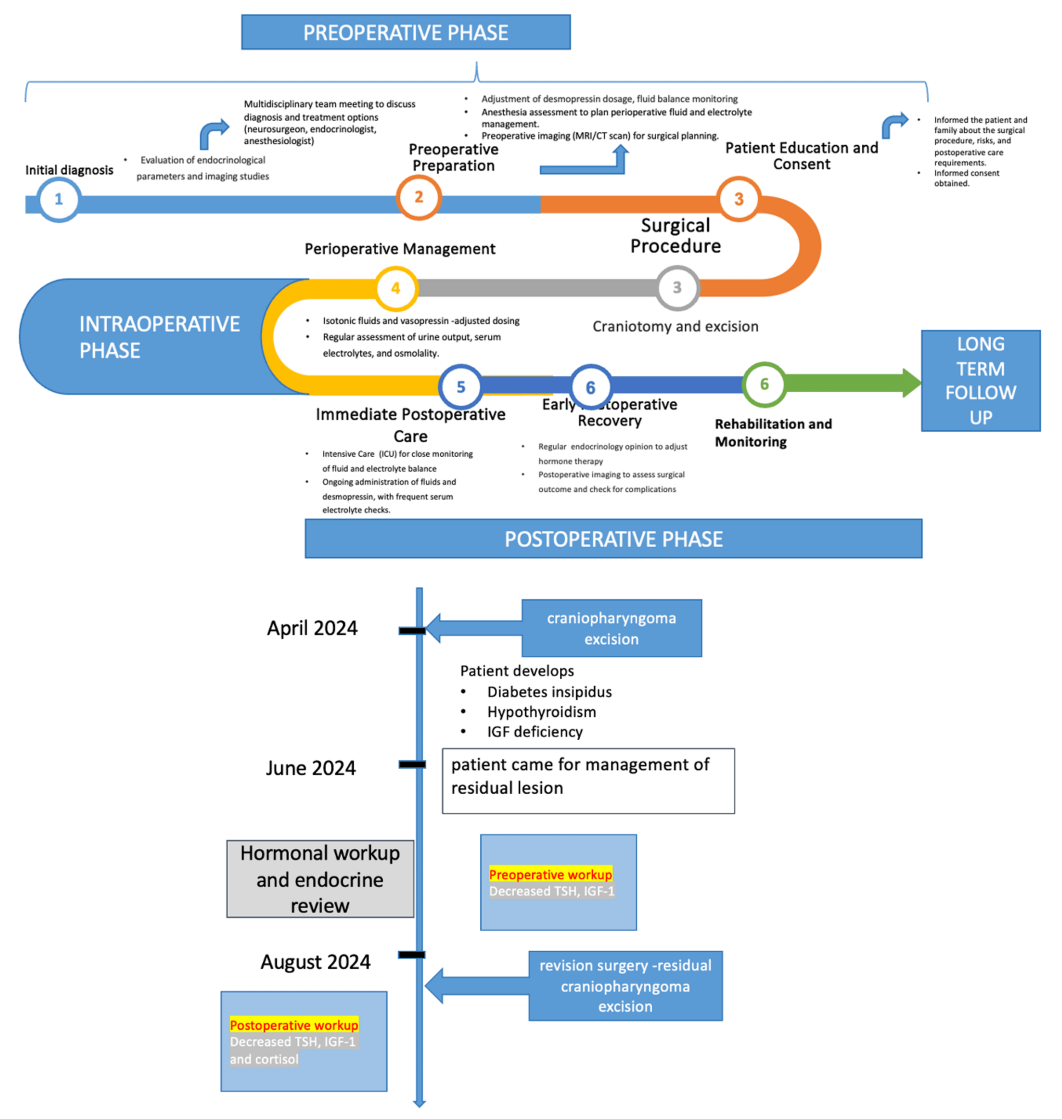
Timeline showing history, laboratory workup and multidisciplinary intervention for patient with residual craniopharyngioma with diabetes insipidus Image credit: Authors. TSH: thyroid stimulating hormone; IGF-1: Insulin-like Growth Factor

Four months later, a repeat radiological scanning revealed a residual craniopharyngioma lesion (Figure 1). Now, she was posted for a redo surgery. The perioperative course of the patient has been summarized in Figure 2. Clinical examination revealed no neurological deficit. The preoperative evaluation involved a comprehensive review of her fluid balance, electrolytes, and endocrine status, with input from the endocrinology team. Emphasis was placed on maintaining adequate hydration and monitoring for potential fluid and electrolyte imbalances. However, the patient was still with central DI with urine output of 3-4 ml/kg/h and a borderline raised serum sodium (Na 145-148 mEq/L), despite being on oral desmopressin. Serum cortisol was within normal limits. Serum TSH (thyroid stimulating hormone) was decreased. However, the serum T3 (triiodothyronine ) and T4 (thyroxine) levels were normal. The rest of the routine laboratory investigations were within normal limits (Figure 2, Table 1).

**Table 1:** Preoperative and postoperative hormone profile of the patient TSH- thyroid stimulating hormone, T3- triiodothyronine, T4- thyroxine, IGF-1- insulin-like growth factor-1

Test	Preoperative value	Postoperative value	Reference level
TSH (µIU/L)	0.082	0.11	0.27-4.20
Serum T3 (ng/ml)	1.55	1.04	0.8-2.0
Serum T4 (µg/ml)	8.09	11.1	5.1-14.1
IGF-1 (ng/ml)	66.50	66.56	170-527
Serum cortisol (µg/dl)	11.3	1.72	4.82-19.5

**Figure 3:**
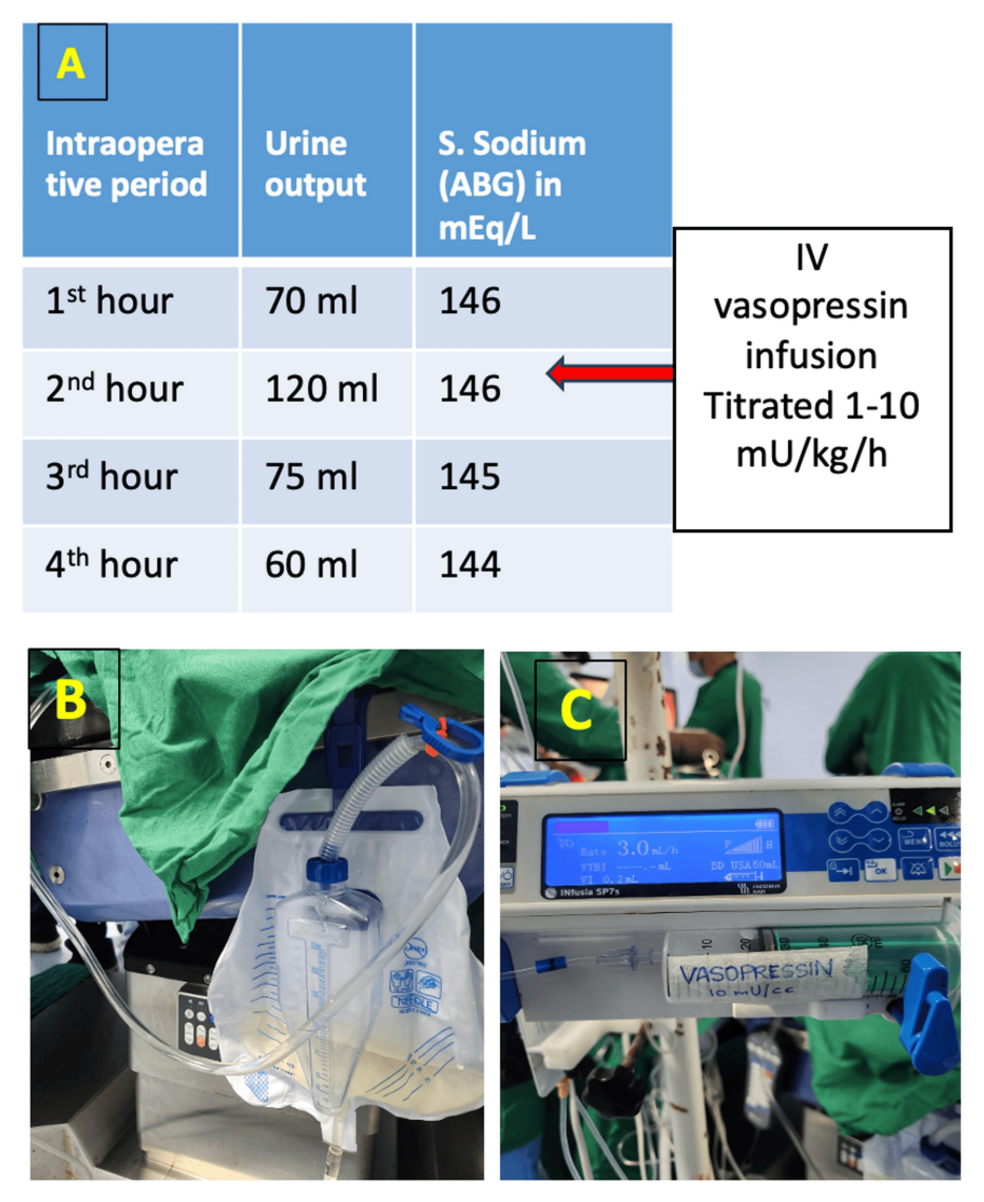
(A) Intraoperative metabolic parameters, (B) Light colored, almost clear urine seen intraoperatively, (C) Low-dose vasopressin (10 mU/ml) infusion ongoing Image credit: Authors.

Intraoperatively, the patient was induced under general anesthesia with fentanyl 70 µg, 70 mg, and atracurium 15 mg. Trachea was intubated with a size 6 cuffed endotracheal tube. Anesthetic maintenance was done with air:oxygen mixture, and sevoflurane. With ultrasound guidance, a 5.5 Fr central venous catheter was inserted in the right internal jugular vein and a 22-gauge left radial arterial line was placed. A nasopharyngeal probe was used for continuous temperature monitoring. Foley’s catheter was inserted and connected to a uroflowmeter bag with gradations. Goal-directed intravenous fluid administration was done with the maintenance of blood gas parameters and normoglycemia. Intraoperatively urine output increased to 70-120 ml/hour during the second hour of surgery, an ABG was done and urine samples were sent. Since the patient already had pre-existing DI on desmopressin and no diuretics were given, a strong suspicion of intraoperative DI was there. Arterial Blood Gas (ABG) revealed serum Na 146 mEq/L and a raised serum osmolarity of 300 mOsm/L. Urine osmolarity was 154 mOsm/L. DI was confirmed (Table 2). An infusion of vasopressin was started in a dose of 1-10 mU/kg/h targeting a urine output of less than 2 ml/kg/h and maintenance fluid shifted to 0.45% normal saline (Figure 3). After starting vasopressin, by the 4th hour urine output had decreased to 2 ml/kg/h and serum Na was 144 mEq/L. Surgically, right pterional craniotomy and near-total excision of the tumor were done. Throughout the surgery, hemodynamics was within 20% of baseline. Postoperatively, the patient was planned for elective ventilation and shifted to ICU on ventilator support. Vasopressin infusion was tapered off within 10 hours as urine output was showing a decreasing trend. Postoperative CT brain was unremarkable. The patient was extubated on postoperative day (POD) 1. The patient was then managed with nasal desmopressin puffs 40 µg SOS. An endocrine review was done. Another significant finding was a decrease in serum cortisol, which was managed with steroid supplementation. Thyroxine supplementation was continued. Her urine output was within normal range (<3ml/kg/h). The patient was shifted out of the ICU on POD 3. The patient’s postoperative course was satisfactory with no neurological deficit and stabilization of DI symptoms. Follow-up advice included routine monitoring for potential tumor recurrence and continued management of endocrine function.

**Table 2:** Intraoperative metabolic parameters

Test	Value	Reference range
Urine osmolarity (mOsm/L)	154	500-800
Urine specific gravity	1	1.005-1.030
Urine sodium (mmol/L)	83.42	-

## Discussion

We reported a rare case of successful intraoperative anesthetic management of a pediatric patient undergoing craniopharyngioma excision with pre-existing central DI. The main anesthetic concerns were a pediatric patient, pre-existing DI, secondary hypothyroidism, intraoperative fluid and electrolyte disbalance, and further risk of hypothalamic damage with the possibility of seizure, hyperthermia, and hemodynamic instability. Patients with DI are at risk for significant electrolyte disturbances due to high urine output and the potential for sodium imbalance. During surgery, maintaining normonatremia and avoiding rapid fluctuations in sodium levels is vital to prevent neurological complications. Desmopressin (DDAVP), a synthetic analog of antidiuretic hormone (ADH), is often used to manage central DI. However, in our case, we chose to give vasopressin infusion in the intraoperative period as it has a faster onset and shorter duration of action (t1/2 10-15 min) contrary to routinely used desmopressin. Also, the enteral and intranasal routes were not feasible in the intraoperative setting. Intravenous low-dose vasopressin is also easy to titrate intraoperatively. A similar case of intraoperative DI managed with vasopressin infusion has been reported [6]. In another study, 18 children either with preoperative DI or undergoing neurosurgical procedures with a high risk for developing postoperative DI were successfully managed using vasopressin infusion [7]. Intraoperative fluid management in a case of DI during craniotomy is more challenging because the massive diuresis and ongoing blood loss together lead to a high probability of hemodynamic disturbances. Intensive monitoring and prompt intervention become necessary in such cases.

Postoperative management usually needs an ICU admission for patients with DI to ensure close neurological monitoring and management of fluid and electrolyte balance. This case report effectively demonstrates comprehensive perioperative management for a patient with central DI undergoing craniopharyngioma revision surgery. The thorough preoperative assessment, including fluid balance and endocrine evaluations, ensured a tailored anesthetic approach. The use of central venous and arterial lines facilitated precise monitoring and fluid management. Collaboration between the surgical, anesthesia, and endocrinology teams was crucial for optimal perioperative management.

## Conclusions

This case report effectively demonstrates comprehensive perioperative management for a patient with central DI undergoing craniopharyngioma revision surgery. The thorough preoperative assessment, including fluid balance and endocrine evaluations, ensured a tailored anesthetic approach. The use of central venous and arterial lines facilitated precise monitoring and fluid management. Collaboration between the surgical, anesthesia, and endocrinology teams was crucial for optimal perioperative management.
